# Gestational Diabetes Mellitus, Breastfeeding, and Progression to Type 2 Diabetes: Why Is It So Hard to Achieve the Protective Benefits of Breastfeeding? A Narrative Review

**DOI:** 10.3390/nu16244346

**Published:** 2024-12-17

**Authors:** María Eugenia Flores-Quijano, Victor Pérez-Nieves, Reyna Sámano, Gabriela Chico-Barba

**Affiliations:** 1 Department of Nutrition and Bioprogramming, Instituto Nacional de Perinatología, Mexico City 11000, Mexico; ssmr0119@yahoo.com.mx (R.S.); gabyc3@gmail.com (G.C.-B.); 2 Faculty of Medicine, Benemérita Universidad Atutónoma de Puebla, Puebla 72410, Mexico; victorpn14@gmail.com

**Keywords:** breastfeeding, gestational diabetes mellitus, type 2 diabetes mellitus, obesity

## Abstract

Women diagnosed with gestational diabetes mellitus (GDM) face a significantly heightened risk of developing type 2 diabetes mellitus (T2DM) later in life. Breastfeeding (BF) has been identified as a potential strategy to delay or prevent T2DM; however, women with GDM often encounter barriers in initiating and maintaining adequate BF practices compared to those with uncomplicated pregnancies. This paradox prompts an exploration into the causes of these BF challenges and considers the possibility of reverse causation: Does prolonged and intensive BF mitigate the risk of subsequent glucose dysregulation and T2DM? Alternatively, do women with compromised insulin secretion and sensitivity, who are predisposed to T2DM, struggle to sustain intensive BF practices? This narrative review aims to explore the interplay between GDM, BF, and T2DM development by examining the different factors that present BF challenges among women with GDM. Understanding these dynamics is crucial for establishing realistic BF expectations and developing effective clinical and public health strategies to support BF in this high-risk population.

## 1. Introduction

The global prevalence of diabetes has reached alarming levels, with an estimated 537 million adults affected in 2021, 90% of whom have type 2 diabetes mellitus (T2DM), a figure that is projected to rise to 783 million by 2045 [1]. Gestational diabetes mellitus (GDM), a condition that affects 16.7% of pregnancies worldwide, significantly contributes to this growing burden [1]. Women with a history of GDM face a 7.4 to 9.6 times increased risk of developing T2DM later in life [2].

Breastfeeding (BF) has been identified as a key intervention to delay or prevent the onset of T2DM due to its positive effects on glucose metabolism and weight regulation [3]. Despite these potential benefits, paradoxically, women with GDM are less likely to initiate and sustain recommended BF practices compared to those without GDM [4]. This discrepancy presents a significant public health challenge, indicating missed opportunities for mitigating long-term health risks in this population.

This narrative review explores the complex interplay between GDM, BF, and the progression to T2DM. The review is structured into two sections. The first provides a comprehensive overview of the protective effects of BF in reducing the risk of T2DM among women with a history of GDM, along with data on exclusive BF rates during the early postpartum period and overall BF duration. The second section examines various factors that pose challenges and barriers to BF for women with GDM. Building on existing research, our discussion explores the possibility of a reverse relationship, where women with GDM may face anatomical, metabolic, or practical challenges that reduce their ability to breastfeed at the recommended intensity and duration.

Ultimately, this article presents a conceptual model elucidating the pathways between GDM and shortened BF duration. It provides a comprehensive understanding of the challenges women face with GDM and serves as a foundation for future research on BF difficulties. Unlike most studies that propose isolate factors—such as BMI or postpartum variables—this model offers an integrated perspective on the pathways through which these factors affect BF [5,6,7].

## 2. Materials and Methods

Multiple literature searches were conducted for this review; PubMed, Scielo, and Google Scholar databases were searched to address the research questions guiding this work. Initially, we searched for articles examining the impact of BF following GDM on the risk of developing T2DM later in life. The search included terms for “gestational diabetes” “breastfeeding” and “type 2 diabetes mellitus”. Since many articles looking at the association between lactation and T2DM incidence in women with prior GDM were systematic reviews and meta-analyses, we prioritized these sources, as they synthesize the available data. Additionally, we focused on the association between GDM and BF rates at hospital discharge and beyond, with particular emphasis on two recent synthesis publications.

In the second section, we focused on identifying studies that explored the factors influencing BF practices and their duration among women with GDM. We categorized these factors into three groups based on the impact of GDM on the mammary gland and lactation physiology: (1) direct factors, which result from glucose dysregulation during pregnancy that may hinder mammary gland development and alter hormone levels; (2) underlying factors, such as obesity, which increase the risk of developing GDM and independently contribute to BF challenges; and (3) indirect factors, which arise from GDM or its complications, indirectly affecting lactation without directly impairing mammary function or hormonal balance. Following this initial search, we conducted targeted database searches and reviewed reference lists to identify additional relevant concepts for each factor. No restrictions were applied regarding the publication date of the included studies.

## 3. Gestational Diabetes Mellitus Progression to Type 2 Diabetes Mellitus and the Role of Lactation

Gestational diabetes mellitus is a pregnancy complication that is diagnosed when women without prior hyperglycemia develop diabetes during the second or third trimester [8]. Its causes are diverse, with some mirroring those of non-pregnancy diabetes. These include immune system attacks on pancreatic β-cells (characteristic of type 1 diabetes), insulin resistance (when the body’s cells respond poorly to insulin, reducing glucose uptake), and β-cell dysfunction (impaired ability of β-cells to produce or release sufficient insulin, characteristic of type 2 diabetes), as well as genetic mutations [9,10]. Approximately 80% of GDM cases closely resemble type 2 diabetes, as they occur against a background of chronic insulin resistance combined with β-cell dysfunction [10,11]. Due to its high prevalence, this form of GDM will be the focus of our review.

During a healthy pregnancy, insulin sensitivity changes to ensure efficient metabolism and proper nutrient distribution between the mother and fetus. In the first trimester, insulin sensitivity increases, enhancing glucose uptake into maternal fat stores to meet later energy demands. From the second trimester, increased fat stores and placental hormones—such as estrogen, progesterone, leptin, and cortisol—reduce insulin sensitivity, leading to a natural increase in insulin resistance. To compensate, insulin secretion increases. These adjustments cause a slight rise in blood glucose levels, which are then transported through the placenta to support fetal growth [12]. However, in some women, this normal increase in insulin resistance overburdens their pancreatic B-cells, which cannot adequately boost insulin secretion. This can lead to hyperglycemia and a diagnosis of GDM. Factors such as having prepregnancy overweight or obesity, experiencing excessive gestational weight gain, having a history of GDM or a family history of insulin resistance and/or diabetes, a Westernized diet, and micronutrient deficiencies further exacerbate insulin resistance and increase the risk of developing GDM [9,11].

While normal glucose metabolism typically returns after pregnancy due to the loss of placental hormones, women with prior GDM often experience a concerningly high prevalence of altered fasting glucose, ranging from 17% to 23% within three months postpartum. Women with abnormal test results face an increased risk of developing T2DM compared to those whose postpartum glucose results are within the normal range [13]. GDM often exposes a hidden metabolic issue that existed before pregnancy and signifies a risk of further metabolic dysregulation and disease progression. Recent meta-analyses, drawing upon extensive cohort studies, have revealed a concerning trend: a 10% higher risk of developing T2DM emerges with every decade of follow-up post-GDM. Projected estimates illustrate this risk trajectory, showing a progression from 19.7% at 10 years to 29.3% at 20 years, 39.0% at 30 years, 48.6% at 40 years, and 58.3% at 50 years, respectively [14].

Breastfeeding is one of the key lifestyle interventions shown to be effective at delaying the onset or reducing the incidence of T2DM among women with a history of GDM [3]. Over the past few decades, numerous observational studies have been conducted to assess the impact of BF on the progression of GDM to T2DM. Various systematic reviews and meta-analyses have synthesized the findings of these studies.

Table 1 provides a comprehensive overview of five published evidence syntheses. While we acknowledge that some meta-analyses include overlapping studies, which may introduce redundancy and potential bias, each synthesis offers unique insights due to differences in how confounders were addressed and in the categorization of independent BF variables. These distinctions are crucial for understanding whether BF, compared to not BF, reduces the risk of developing T2DM, as well as for evaluating how variations in BF duration and intensity influence the incidence of T2DM.

Three meta-analyses, which exclusively focused on women with prior GDM, revealed that lactation, compared to non-lactation, provided an overall protective effect ranging from 34% to 52% against the development of T2DM [15,16]. Subgroup analysis by Feng et al. demonstrated that this protective effect persisted irrespective of the cohort studies’ temporality (prospective or retrospective). However, the effect was deemed non-significant in cases where the sample size exceeded 500, the follow-up duration was less than a year, or the meta-analysis utilized unadjusted data [17].

Concerning BF duration and the incidence of T2DM after GDM, four meta-analyses investigated the relationship [16,17,18,19]. Among them, three identified a protective effect associated with prolonged BF practice. Two studies used a dichotomous categorization of lactation into short and long durations using arbitrary cut-off points. For instance, the meta-analysis by Ma. et al. included 15 studies and used a relative length for each included study. In comparison, the meta-analysis by Tanase-Nakao used a standardized definition for the five included studies (<4 to 12 weeks postpartum for short and >4 to 12 weeks for long duration). Both analyses demonstrated a reduced risk of T2DM progression. Notably, both agreed that this protective effect was absent when the follow-up was shorter than six months and became more pronounced with a follow-up period longer than one year [18,19]. Interestingly, Ma et al. conducted a subgroup analysis revealing that the protective effect of longer lactation was absent when only cross-sectional studies were pooled [18]. Pinho-Gomes et al. performed a dose–response meta-analysis, which overcame the limitations of the dichotomic categorization, and found that each additional month of lactation was associated with a 1% reduction in T2DM risk [16]. The fourth meta-analysis, encompassing three studies, found no protective effect for T2DM in the category of long-term lactation. This analysis pooled the data and compared >4 to 12 weeks of lactation with no lactation [17].

Two meta-analyses included information regarding BF intensity, each analyzing information from only one or two studies. Both reviews found a significant protective effect against progression to T2DM. Ma et al. [18] reported a 47% reduction in risk, while Tanase-Nakao et al. [19] found a 58% decrease in risk among women who practiced full or exclusive BF, respectively.

The published literature consistently demonstrates that BF seems to have a protective effect on GDM progression to T2DM. The longer and more intensive the practice, the stronger the protection conferred.

Various protective mechanisms against T2DM progression have been proposed. For instance, during lactation, changes in glucose homeostasis are observed, characterized by increased non-insulin-dependent glucose uptake by the mammary glands. This uptake potentially induces B-cell rest by reducing insulin production [20]. Furthermore, there is evidence of increased B-cell mass and function during lactation. This enhancement in B-cell mass and function is believed to contribute to improved glucose homeostasis, which lasts for years after the cessation of lactation [21]. In addition to the mechanisms that directly concern an improvement in glucose metabolism, BF has been associated with higher metabolic expenditure, an increased rate of lipolysis, and less weight retention after pregnancy, which are risk factors for T2DM. Furthermore, lactation practice has been associated with other healthy behaviors [16,22] that may protect against glucose dysregulation.

However, based on the available evidence from observational studies, reverse causation cannot be ruled out. Does longer and more intense BF reduce the risk of further glucose dysregulation or T2DM? Alternatively, could women with greater impairments in insulin secretion and sensitivity—those who are more likely to develop T2DM—be facing greater challenges in maintaining prolonged and intense BF practices? [19,22,23].

Temporality [24]—the concept that BF precedes the development of T2DM—remains difficult to establish due to the observational nature of most BF studies. Randomizing BF behavior is not feasible due to ethical and practical considerations. Even in prospective cohort studies, where BF occurs first and its duration is tracked before monitoring diabetes outcomes, unaccounted confounding variables [19] in individual studies or meta-analyses may obscure the directionality of findings.

For instance, some characteristics of GDM may increase the risk of developing diabetes after pregnancy, independent of lactation practices. A notable example is the presence of positive islet antibodies, which significantly heighten the risk of developing type 1 diabetes, regardless of BF, a factor that is not controlled for in some studies [10,25]. With respect to T2DM, the severity of GDM—often categorized by the type of treatment required, whether managed through diet, oral hypoglycemic agents, or insulin—[26] and the degree of insulin resistance, βcell-dysfunction and the presence of obesity are all independent factors that could influence the risk of developing T2DM later in life [9].

Moreover, certain pathophysiological traits of GDM, such as insulin resistance and the limited capacity of β-cells to increase insulin secretion, may be latent before pregnancy, becoming apparent due to the metabolic demands of gestation. GDM is thus sometimes considered a stage in the progression of diabetes [9]. These factors add complexity to understanding the temporality and interplay between GDM, BF, and the progression to T2DM, creating additional uncertainties about their relationship.

The possibility of reverse causality highlights that the factors increasing diabetes risk may also hinder lactation outcomes. This could, at least partially, explain the consistently lower prevalence of recommended BF practices among women who experienced GDM during pregnancy, as documented in various studies.

## 4. Breastfeeding Prevalence and Duration Among Women with GDM

Despite the known benefits of lactation after the diagnosis of GDM, two recent evidence synthesis publications documented that this health condition negatively influences BF prevalence and duration. The first is a systematic review that includes studies published from 1985 to 2017. It consisted of 16 low- to medium-quality studies, mainly from the US (*n* = 11) and other developed countries (*n* = 5) [27]. Regarding prevalence rates, four studies compared the rates of exclusive or predominant BF during early postpartum hospitalization, and all of them (four out of four studies) observed a lower rate among women with GDM. However, in three studies where the observed outcome was “any BF” at hospitalization discharge, there was no difference between the GDM groups (three out of three studies). Concerning BF duration, two out of three studies comparing the extent of exclusive or predominant BF after hospital discharge found shorter lengths among women with GDM. It is worth mentioning that the studies that found a difference in the duration of exclusive BF or predominant BF between the GDM status groups used a continuous variable, which offers a more nuanced measure. These studies observed a duration of 2 [28] and 9 [29] weeks in the GDM group compared to 3 and 17 weeks in the non-GDM groups. In contrast, the third study, which found no difference between the groups, used a categorical variable comparing the proportion of women exclusively or predominantly BF at <12 or ≥12 weeks [30]. Such a broad category might have missed differences present in the earlier postpartum weeks. In the systematic review, most studies (8/11) comparing the length of “any BF” found no difference between the groups.

The second publication is a meta-analysis, including 22 very low to low-quality studies from 18 developed (12 studies) and developing (6 studies) countries, some of which were included in the aforementioned systematic review. This analysis, published in 2020, endorses the previous finding that women with GDM are more likely to utilize formula milk before hospital discharge (OR 1.36; 95% confidence interval (CI) 1.22–1.51, *p* < 0.001). However, the meta-analysis comparing mothers with and without GDM found similar rates of exclusive or predominant BF at ≥5 months (19.5% vs. 21.0%, OR 0.89, 95% CI 0.79, 0.01, *p* = 0.07), lower rates of continued BF at 12 months (65.2% vs. 73.7%, OR 0.66 95% CI 0.51–0.85, *p* = 0.002), and a shorter BF duration (months) (standardized mean difference −0.19; 95% CI −0.26, −0.12, *p* < 0.001) [4]. As noted in the previous paragraph, regarding the difference in the prevalence of EBF and PBF between GDM groups, the cut-off point of 5 months may be too late to observe a possible difference during the first few weeks and months postpartum.

Several observations have emerged regarding the influence of GDM on BF prevalence and duration:

1. During early postpartum, women diagnosed with GDM exhibit a heightened likelihood of discontinuing (or not starting) exclusive BF, often introducing formula milk before leaving the hospital. However, the consistency of differences in “any BF” rates between GDM and non-GDM groups upon discharge is less consistent.
2. Following hospital discharge, women with GDM tend to sustain exclusive or predominant BF for shorter durations compared to their counterparts without GDM. Moreover, they demonstrate lower rates of continued BF at the 12-month mark, resulting in shorter BF durations.
3. Published studies vary in terms of the methodologies employed to measure BF rates and duration, which impacts our ability to discern the influence of GDM. Studies utilizing continuous variables have been more adept at detecting disparities in exclusive BF duration than those utilizing categorical variables with broad cut-off points, such as <12 or ≥12 weeks for early BF and ≥5 months for later stages. This discrepancy may potentially underestimate the impact of GDM on early BF practices.

These observations contribute to the larger narrative of this review, highlighting the complex interplay between GDM, breastfeeding challenges, and the increased risk of developing T2DM. While women with GDM initiate breastfeeding at rates similar to those without GDM in the early postpartum period, they often face difficulties in maintaining exclusive breastfeeding (EBF) and are more likely to introduce formula before hospital discharge. Furthermore, women with GDM tend to discontinue breastfeeding earlier, resulting in shorter durations of both EBF and any breastfeeding.

This raises an important question: What specific factors associated with GDM prevent women from adhering to recommended breastfeeding practices, potentially increasing their risk of progressing to T2DM?

In the following sections, we explore the potential contributing factors that explain these breastfeeding challenges, providing a deeper understanding of this phenomenon.

## 5. Factors Contributing to Challenges in Breastfeeding Among Women with GDM

**Figure 1:**
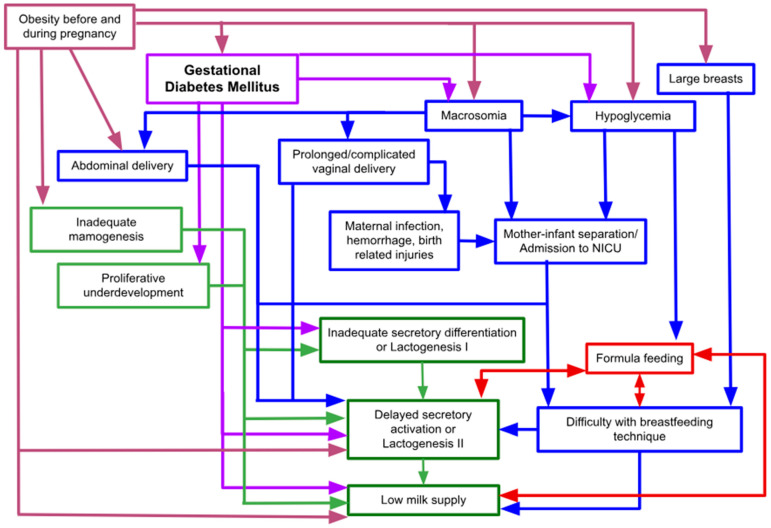
Direct, underlying and indirect factors for breastfeeding disruption among women with GDM. GDM (framed in purple) serves as the precursor to several direct factors (framed in green) contributing to BF difficulties, including altered proliferative and secretory phases of mammary gland development during pregnancy, delayed secretory activation, and low milk supply. Additionally, GDM increases the risk of indirect factors (framed in blue) such as macrosomia and hypoglycemia, which impact BF indirectly. Underlying factors, such as prepregnancy obesity (framed in pinkish-tan), may induce inadequate mammogenesis—a direct factor in BF difficulties (framed in green)—and may also increase the risk of GDM (framed in purple), and prepregnancy obesity is often associated with larger breast size (framed in pinkish-tan). Large breast size can complicate BF technique (framed in blue), potentially triggering delayed secretory activation and a low milk supply (framed in green). In addition to GDM, prepregnancy obesity also precedes indirect factors (framed in blue) contributing to BF difficulties, thereby causing predisposition to direct disruptive factors (framed in green), mediating other indirect factors, or even encouraging patients not to breastfeed and to introduce formula instead (framed in red).

The negative effect of GDM on BF practice and its length may be understood by dividing the possible factors into direct, underlying, and indirect categories (Figure 1), according to how directly GDM affects the mammary gland and lactation physiology.

### 5.1. Direct Factors

The direct factors of inadequate BF stem from the pathophysiological effects of glucose dysregulation during pregnancy, which can impede the adequate anatomical development of the mammary gland and alter the hormonal milieu (Figure 1). Therefore, it impacts the timely onset of copious milk production after delivery and the synthesis rate of milk during established lactation.

To better understand this, we may explain the development of the mammary gland during pregnancy in two phases. The first phase consists of the proliferation of glandular tissue during early pregnancy, in which mammary epithelial cells grow and multiply to form new alveoli (alveologenesis) and to expand ductal branches from the terminal lobular ductal units near the nipple into the mammary stroma. The second phase, known as secretory differentiation or lactogenesis I, begins by mid-pregnancy when the proliferation has decreased significantly, and the alveolar progenitor cells transform into functional milk-secreting alveoli [31,32].

Insulin signaling has been identified as a mediator in mammary development’s proliferative and differentiation phases. Consequently, insulin dysregulation may impede the potential growth and development of the gland, as well as the timely onset and rate of milk production [32]. In a study involving mice with reduced expression of insulin receptors during gestation, the mammary glands showed smaller size and a 50% decrease in alveoli at mid-pregnancy. Consequently, these mice produced 60% less casein and 75% fewer lipid droplets, indicating impaired mammary secretory differentiation and inadequate preparation for secretory activation. By day 18 postpartum, coinciding with the peak lactation stage, the pups exhibited notably slow growth rates, suggesting insufficient milk secretion [32]. However, apart from the potential impact of mammary gland underdevelopment on milk secretion, the diminished litter growth rate may also be attributed to the role of insulin and its interaction with the insulin receptor in the biosynthesis of milk components and the maintenance of milk secretion during established lactation. For example, in vitro studies using bovine and murine mammary explants have shown that insulin stimulates the expression of genes involved in milk protein, lipid, and lactose synthesis [32,33,34]. Furthermore, the developmental process of the mouse mammary gland during pregnancy mirrors the remodeling undergone by the human gland during this stage [35]. Therefore, insulin likely plays a significant role in the gestational development of human mammary gland alveoli structures and milk synthesis, as supported by in vitro mammosphere model studies using human mammary epithelial cells [36].

Several studies have evidenced the functional consequences of glucose dysregulation during human pregnancy, from birth onward. Such consequences include delayed secretory activation or lactogenesis II and low milk supply during BF. Regarding delayed secretory activation, defined as the initiation of copious milk production after 72 h postpartum, frequently assessed by the maternal perception of breast fullness, is 1.84 times more likely to occur among women with GDM than in women without GDM, according to a meta-analysis of eight studies [37]. Furthermore, the severity of gestational diabetes has also been found to be an important predictor of delayed secretory activation. A cohort of women with GDM documented that the use of insulin as a treatment for GDM—which is an indicator of the most severe form of the disease—compared to treating GDM with diet alone was associated with a much higher risk (OR: 3.11; 95% CI 1.37, 7.05; *p* = 0.0076) of delayed secretory activation [26]. This observation was confirmed in a later meta-analysis (OR = 3.07, 95% CI 1.71–5.47) [37]. The documented adverse consequences of experiencing delayed secretory activation are an increased risk of excessive neonatal weight loss (≥10% of birth weight) in the early postpartum period [38,39], a shorter duration of exclusive and any BF [23,40,41], as well as self-reported insufficient milk [42], which may imply a low milk supply.

In fact, low milk supply, defined as insufficient milk production despite the absence of latch and/or nipple problems, has been characterized as a biological consequence of GDM over lactation. A case–control study designed to determine the association between diabetes (not specifically GDM) in pregnancy and low milk supply in women around the third to sixth weeks postpartum, controlled by several confounding and mediating factors such as maternal history of polycystic ovary syndrome, delivery mode and prematurity, found an independent 2.6-fold greater odds of reduced milk volume among women with impaired glucose intolerance [43]. A low milk supply has been observed in women with GDM despite regular milk removal, which is suggested to facilitate sufficient milk production [44]. Another study used milk concentrations of sodium (Na) and the Na:K ratio as indicators of low milk supply. It observed a positive association between fasting plasma glucose and both Na (adjusted coefficient 1.28, 95% CI 1.08–1.51) and the Na:K ratio (1.29, 95% CI 1.08–1.54) in women 3 weeks postpartum [45]. However, the models did not adjust for BF intensity (exclusive, full, or mixed), which could potentially influence these electrolyte concentrations [46].

To sum up, the pathophysiological impacts of glucose dysregulation during pregnancy can impede multiple stages of lactation. These effects include the inhibition of mammogenesis in early pregnancy, impaired lactocyte differentiation in late pregnancy, delayed lactation onset, and a reduced rate of milk production [23]. Additionally, the severity of glucose dysregulation has been shown to significantly impact BF difficulties, particularly increasing the risk of delayed secretory activation. This contributes to the outgoing discussion about the possibility of reverse causality: more severe dysregulation not only raises the likelihood of delayed lactation onset but also leads to a greater probability of not practicing EBF. In turn, this may increase the risk of developing T2DM through two pathways: the already compromised glucose metabolism, which progresses to GDM independently of BF, and the reduced protective effects of optimal BF practices. These findings underscore the intricate interplay between GDM, BF challenges, and long-term metabolic health outcomes.

### 5.2. Underlying Factors

The underlying factors of disruptions in BF encompass factors that not only heighten the likelihood of developing GDM but also act as independent risk factors for BF failure. These factors include a high pregestational body mass index (pBMI), the consumption of a high-fat or “obesogenic diet”, polycystic ovary syndrome, or a history of previous GDM.

High pBMI is prevalent among childbearing-age women worldwide and is a significant contributing factor to BF challenges. Pregnant women with a high pBMI face a risk of abnormal glucose metabolism that is at least twice as high. This heightened risk follows a dose–response pattern: pregestational overweight doubles the likelihood of developing gestational diabetes (OR 1.97 to 2.01 from different studies), moderate obesity triples it (OR 3.01), and morbid obesity results in a fivefold increase (OR 5.55) [23,26,47,48]. Furthermore, women classified as overweight or obese face an increased likelihood of encountering difficulties in prompt initiation of BF, as well as experiencing shorter durations of exclusive and continuous BF [48,49,50,51]. Obesity and GDM together compound breastfeeding difficulties, as both conditions exacerbate insulin resistance and disrupt mammary gland development. This overlap may increase the risk of breastfeeding failure and progression to T2DM.

High pBMI and the other underlying factors impact BF through two distinct mechanisms: the direct pathway, marked by a shared or even potentiating role of the pathophysiological mechanisms through which GDM negatively affects the anatomy and functionality of the mammary gland and physiology of lactation, and the indirect pathway, which encompasses factors associated with the underlying causes of GDM, independent risk factors for BF challenges. Examples of these factors include large breasts that may hinder effective infant latching, cesarean delivery, preterm birth, macrosomia, and hypoglycemia [52]; these will be discussed in the coming section.

Through the direct pathway, excess adiposity impacts lactation in two dimensions. First, it exerts a morphological influence at different stages of life, altering the anatomy and functionality of the mammary gland and potentially limiting its full milk production capability. Second, excess adiposity detrimentally affects endocrine regulation, influencing the physiology of lactation.

The impact of obesity on mammary gland morphology may occur during crucial developmental stages such as puberty, when terminal end buds, ductal elongation, and branching take place, or later during pregnancy, when mammary structures proliferate and differentiate [31,53]. Studies conducted in mice, rabbits, and dairy cattle have highlighted the effects of obesity or consumption of an “obesogenic diet” during critical periods of mammary morphogenesis. However, conclusive findings are complicated by factors such as variations in breed or strain [54], interspecies differences [55,56], genetic diversity, and the timing of exposure to obesogenic diets in relation to age or life stage [55,56]. Furthermore, the specific composition of the high-energy diet plays a crucial role [56]. Nonetheless, significant overarching observations emerge, highlighting that the type of negative effect differs depending on the developmental stage at which obesity or high nutritional intake occurs. When a nutritional assault occurs before and during puberty, it is associated with enlarged yet underdeveloped mammary glands. These glands exhibit increased adiposity; stunted, less dense, and branched ducts; along with less developed alveoli [54,56,57,58,59]. Importantly, these morphological differences persist during pregnancy and lactation, ultimately negatively impacting milk yield [60]. In contrast, high levels of feeding during adulthood and pregnancy do not yield significant effects on the proliferation of mammary gland structures [54,61].

Nonetheless, the study by Flint et al. documented impaired alveolar differentiation into secretory mammary epithelial cells or lactocytes, suggesting a potential delay in secretory activation and subsequently lower milk production, illustrated by a decreased pup weight gain during the first day of lactation. In this study, pup weight gain was alleviated during the following 2 to 3 days. However, as the authors point out, impaired secretory activation in women with obesity is associated with the early introduction of other milk and with early BF cessation [61].

Beyond the morphological impact of obesity on the anatomy and functionality of the mammary gland, excess body fat during lactation disrupts endocrine regulation and detrimentally affects the physiology of lactation.

In humans, the delivery of the placenta following childbirth results in a rapid decline in circulating progesterone levels and triggers the secretory activation phase within 30 to 40 h post-delivery [47]. Estrogen concentration also decreases after placental delivery, while the presence of hormones like prolactin is essential for achieving secretory activation. Additionally, hormones such as oxytocin play a crucial role in initiating and maintaining milk production. However, as discussed below, obesity disrupts hormonal regulation and poses challenges to lactation.

If serum progesterone levels remain elevated—for example, when placental fragments are retained—lactation will not be fully established. In this context, two studies compared serum progesterone concentrations between obese and non-obese individuals. The first found no difference at the 37th week of gestation and 48 h postpartum [62], while the second found no difference at 48 h and 7 days postpartum [63]. However, this hypothesis cannot be dismissed yet, as suggested by other authors [53,64]. It is plausible that 48 h postpartum might be too early and 7 days postpartum too late to detect group differences. Additionally, higher progesterone concentrations have been observed in the adipose tissue of women with obesity compared to those with normal weight [65]. This implies that mammary fat pads may sequester progesterone in adipose tissue during lactation and, theoretically, delay secretory activation. Further studies are required to compare progesterone concentrations in serum across BMI groups in the early stages of lactation and establish its potential effect on milk production. Additionally, it is necessary to determine whether the mammary gland responds to local progesterone in breast fat tissue [53].

Estrogen is another hormone that suppresses milk production when present in high concentrations during lactation [64]. A prospective observational study that followed 91 women for 6 months postpartum found a negative association between blood estradiol and milk output at 1 month of age [66].

In individuals with obesity, adipose tissue produces estrogen, particularly estrone, due to the presence of the enzyme aromatase, which converts androgens into estrogens. This process may be further facilitated by increased adrenal secretion of androgens in subjects with obesity [67]. Notably, estrone is the major estrogen metabolite in milk [68], and it has been proposed that extragonadal estrogens are only biologically active in the tissue where it was produced (intracrine) [69]. Therefore, further investigation is required to understand the relationship between obesity, mammary fat pad estrogen production, and lactation [53].

As previously mentioned, prolactin is crucial in facilitating the secretory activation phase of lactation. Studies conducted on mice have revealed that this hormone promotes the closure of tight junctions between alveolar cells, which is essential for initiating copious milk secretion while enhancing the expression of genes involved in milk synthesis [31]. In women with obesity, two proposed mechanisms regarding prolactin action aim to explain its potential involvement in impaired lactation performance.

The first mechanism, observed in animal studies, suggests a blunted action of prolactin, or prolactin resistance, which may result from elevated levels of circulating leptin produced by adipose tissue. Research on mice with obesity subjected to a high-fat diet has shown that leptin receptors are present in prolactin-responsive cells within both the mammary gland and the hypothalamus. Elevated leptin levels have been found to diminish the mammary gland’s response to prolactin stimulation, leading to decreased milk production. Notably, this reduction in milk production was observed not during the early postpartum period when milk production is lower, but when there is a higher volume demand. Prolactin resistance was further substantiated by findings indicating lower calorie intake and weight reduction among mice with obesity around the same time as lactation when milk production was different between groups, suggesting that leptin is not blunted by prolactin and thus may exert an anorexic effect [70]. In women, there are no studies relating prolactin resistance to obesity; this is a hypothesis that deserves to be further studied.

The second mechanism observed in human studies is a lower prolactin response to suckling, defined as the difference between basal and post-suckling prolactin concentrations during early lactation. A study comparing prolactin excretion between normal-weight and women with overweight/obesity observed, after adjusting for confounders, that the prolactin response to suckling was lower in the overweight/obese women at days 2 and 7 postpartum [63]. However, another intriguing finding of this study is that leptin was negatively associated with the prolactin response to suckling at seven days postpartum. Other studies have documented a negative association between leptin and prolactin at 37 weeks of gestation, day 2 postpartum [62], and at 3 and 6 months postpartum [71]. A negative relationship between leptin and milk volume has been observed, and could potentially be explained by the association between the two hormones [71]. The authors suggest that excessive leptin concentrations inhibit prolactin secretion. In these human studies, it seems that the relationship between leptin and prolactin is mediated by obesity, and the relationship between leptin and milk volume is mediated by prolactin.

It would be interesting to integrate these two possible mechanisms and understand whether they are indicative of a relationship between the concentration of the hormones leptin and prolactin or an effect of resistance to prolactin produced by excess leptin.

Finally, oxytocin, a hormone responsible for stimulating the contraction of myoepithelial cells surrounding the alveoli, is crucial in facilitating milk ejection into the milk ducts during BF. However, obesity may also interfere with this hormone and hinder lactation. In the context of uterine physiology during pregnancy, progesterone binds to oxytocin receptors, effectively reducing the sensitivity of the uterus to oxytocin. This mechanism ensures the maintenance of uterine quiescence [72]. Also, some proinflammatory cytokines have been shown to down-regulate oxytocin receptors in uterine cells [73]. For example, an in vitro study using biopsies of human myometrium observed that excessive leptin exerted an inhibitory effect on oxytocin-induced uterine contractions and possibly explained dysfunctional labor and increased rates of cesarean birth among obese women [74]. In accordance with this observation, studies in mice have proved that leptin might inhibit oxytocin secretion by lowering noradrenergic neurotransmission in the paraventricular nuclei in the hypothalamus [75]. In the lactation context, excessive progesterone or leptin may inhibit the adequate contraction of the alveolar cells, thus reducing milk transference into the ducts and its availability. In mice, myoepithelial cells have been described as the major population of leptin-responsive cells in the mammary gland, suggesting leptin may interfere with oxytocin action through its direct action on these cells [70]. These mechanisms could further be explored in the context of lactation in women with obesity and other metabolic dysregulation, such as gestational diabetes.

In summary, the presence of obesity in earlier stages of life increases the risk of developing GDM and potentially amplifies the negative effects of GDM on BF practices. At the same time, obesity independently increases the risk of developing T2DM later in life, regardless of BF status [14]. This scenario exemplifies one of the complexities in interpreting the temporality of the relationship between BF and the development of T2DM. This complexity arises because (1) factors that hinder BF, such as the potential underdevelopment of the mammary glands’ morphology and functionality due to obesity, may precede the development of GDM; and (2) the negative effects of GDM on BF are compounded by the hormonal changes induced by excessive body fat, which further disrupt the physiology of milk production.

### 5.3. Indirect Factors

Indirect factors can also influence lactation disturbances among women with GDM. These factors are deemed “indirect” because they arise as a result of GDM occurring during pregnancy or from underlying conditions such as obesity. Although they do not directly impair the function of the mammary gland or alter the hormonal environment essential for milk production, these factors can still negatively impact lactation (Figure 1). Neonatal complications such as macrosomia and hypoglycemia, which are often interrelated and commonly associated with GDM and obesity, can negatively affect lactation by contributing to complications or medical interventions that impede the early and effective initiation of BF [76]. Macrosomia, for instance, may lead to prolonged and complex vaginal deliveries, elevating the risks of maternal infections, postpartum hemorrhage, and birth-related injuries for both the mother and the infant. Furthermore, it is closely linked to an increased likelihood of cesarean deliveries, which may result in postoperative challenges, including incisional pain and reduced mobility [76,77,78]. These complications may disrupt maternal–infant bonding [79] and delay the onset of milk production (secretory activation) [78], further complicating BF initiation. Additionally, hypoglycemia, along with perceived low milk supply, has been identified as a primary reason for the use of formula supplementation during hospital stays among women with GDM [80].

Another BF challenge that arises as a result of a high BMI is the presence of a large body and breast size [50]. This anatomical feature can present practical and mechanical challenges when it comes to positioning the baby and achieving a proper latch [81]. Clinical observations have noted difficulties in positioning the baby and the baby’s reluctance to latch onto the breast [82,83,84]. However, studies have not consistently found an association between large breast size and latching difficulties. Some studies reported small sample sizes [50], while others indicated that women with obesity were more likely to discontinue BF due to “BF difficulties” without specifying the nature of these difficulties [85]. Additionally, a study grouping women with “abnormally large breasts” with other breast variations such as large, flat, or inverted nipples did not specifically identify an association between breast size and a problematic BF technique [86].

Lastly, certain cognitive factors, such as the intention to breastfeed—which is influenced by knowledge, beliefs, expectations, and self-efficacy regarding BF—are also indirect factors that may affect BF practices [80], particularly among women with GDM, both with and without obesity as an underlying factor. In studies comparing the intention to breastfeed between women with GDM and those without, the results have been mixed. One study documented a lower frequency of prenatal intention to breastfeed among women with GDM [87], while other studies found no significant differences between the groups [88,89,90], and some even observed a trend toward a stronger intention to breastfeed in women with GDM [91]. However, the presence of obesity has consistently been associated with a lower intention to breastfeed [92].

As expected, the lack of intention to breastfeed has been associated with a reduced likelihood of exclusive BF in the immediate postpartum period, as well as a shorter overall BF duration [5,87,93]. More notably, studies have shown a tendency for women with GDM [89,90,91] and those with obesity [94] to fail in fulfilling their BF intentions. For instance, one cohort study involving 29 women with GDM and 28 without GDM demonstrated that women with GDM had a higher probability of not meeting their BF intention (OR 8.0, 95% CI 1.89–33.85) and not exclusively breastfeeding (OR 17.4, 95% CI 3.28–92.61). This failure to fulfill BF intentions was associated with neonatal hypoglycemia and admission to the NICU [91]. These findings suggest that although cognitive factors such as BF intentions are influential, other factors—such as pregnancy outcomes—may have a more significant impact on BF practices.

In conclusion, the indirect factors influencing lactation in women with GDM are diverse and multifaceted, encompassing neonatal complications, surgical interventions, hospital practices, anatomical challenges, and cognitive factors. While these elements do not directly impair milk production or hormonal function, they create substantial barriers to successful BF. These barriers often lead to delayed initiation, early formula introduction, and shorter BF durations.

Regarding the interplay between GDM, BF, and the progression to T2DM, indirect factors work in conjunction with the physiological effects of GDM and obesity, further complicating BF. For example, a woman with GDM already faces a higher risk of delayed secretory activation. When combined with a cesarean section—another factor that disrupts secretory activation—the overall risk may be amplified. These combined factors reduce the likelihood that BF will provide protective effects against the progression to T2DM. However, it is important to note that women who experience one or more indirect factors may already be at a higher risk of progressing to T2DM, regardless of their BF practices. Further research is needed to better understand the intricate relationships between these factors and the long-term health outcomes in women with GDM.

## 6. Discussion

Breastfeeding offers numerous health benefits for mothers, including improved glucose homeostasis, which can help protect against the progression to T2DM in women who experienced GDM. Studies highlight that the benefits of BF are closely tied to its intensity [18,19] and duration [16,18,19]. To ensure that both mothers and their babies fully benefit, current recommendations emphasize initiating BF within the first hour after birth, practicing rooming-in, and exclusively providing breast milk for up to six months, followed by continued BF alongside complementary foods for up to two years or beyond [95].

However, these recommendations assume that all women are biologically capable of optimal BF, and when this is not achieved, challenges are often attributed to social or behavioral factors [45,96]. A paradox emerges with women who face complications like GDM, as they tend to discontinue BF earlier and for shorter durations.

It is important to recognize that metabolic disorders such as GDM disrupt BF by influencing factors at different levels—direct, underlying, and indirect—that can potentially affect everything from the morphology of the mammary gland to the hormonal environment that influences the adequate and sufficient production of milk, as well as practical issues that hinder correct BF practice and technique.

Upon reviewing various factors that challenge BF in women with GDM, we hypothesize that disruptions in BF can vary in severity. Factors may accumulate or interact, resulting in different levels of BF impairment. For example, a woman with GDM who is able to manage her glucose levels through dietary changes (non-severe form of metabolic dysregulation) is likely to have better BF outcomes and benefit from the metabolic regulation effects of BF compared to a woman who has been obese since puberty, developed GDM requiring insulin treatment (a severe form of dysregulation), and then underwent a cesarean delivery. She may struggle to achieve optimal lactation and, consequently, may be less likely to derive the benefits of BF. This is due to her suboptimal BF practice and the likelihood that severe metabolic dysregulation outweighs the potential beneficial influence of lactation on glucose and metabolic regulation.

## 7. Conclusions

Recognizing that certain women—such as those with GDM—face greater challenges in achieving optimal BF is crucial. Acknowledging these differences offers an opportunity to tailor interventions, support, and counseling to meet women’s specific needs. Based on the observations and conclusions of this review, we recommend that future work focuses on analyzing proposed interventions for breastfeeding in women with GDM. Evaluating what has been successful and what has not will be essential for developing effective clinical management strategies to help these women reach their full BF potential and maximize the associated health benefits.

## Figures and Tables

**Table 1:** Systematic reviews and meta-analyses on the association between lactation and T2DM incidence.

Year, Type and Aim	Search Strategy	Studies	Variable Definition	Meta-Analysis	Observations
**Pathirana et al., 2022 [15]** SR and MA of observational studies To determine the effects of BF on CV risk factors (including T2DM) in women with previous GDM.	Medline, CINAHL, and Embase From inception to May 2020 English language only	Seven studies which only included women with prior GDM and informed about T2DM. All from high-income countries All cohorts	GDM IADPSG or other previously accepted definition Different definitions of BF used among studies It is not specified if adjusted or unadjusted data were used in the meta-analysis	**BF vs. no-BF** **Four studies** Not breastfeeding increased the risk of T2DM compared to that in women who breastfed RR 2.08; 95% CI 1.44, 3.00, *p* < 0.001; *I*^2^ = 0% (reciprocal RR 0.48; 95% CI 0.33, 0.69) In the MA, two out of four studies had a F/U of <3 m, while the other two had 2 and 7 years. Sample sizes were <50 (1 study) and ≈500 to 1000 (3)	Three studies not included in the MA Two studies reported that BF is associated with a reduction in T2DM In one study, BF had no effect on the progression to T2DM
**Pinho-Gomes AC et al., 2021 [16]** SR and MA of observational studies To investigate the effects of BF on maternal risk of T2DM overall and, according to previous history of GDM, the existence of a dose–response relationship between BF and maternal risk of T2DM.	Medline and Embase From inception to Feb 2021 Search terms provided No type of study or language restriction.	Eleven studies conducted analyses including only women with GDM; five of these were included in the MA All participants were from high-income countries, with representation of ethnic minorities. All cohorts: 6 prospective and 7 retrospective	Different definitions of BF used among studies All studies adjusted at least for age and BMI, with most adjusting for other variables such as ethnicity, education, smoking, and parity	**BF vs. no-BF** **Five studies** BF reduced the risk of T2DM compared to no-BF BF is associated with a 34% lower risk of T2DM RR 0.66, 95% CI, 0.52, 0.85, **Duration of lactation** **Dose–response** **Five studies** Each additional month of lactation was associated with a 1% lower risk of T2DM. RR 0.988, 95% CI 0.981, 0.994 The F/U of the included studies were 2 y (2 studies); 7 y (1); 19 y(1); and 24 (1). Sample sizes were 200–500 (3); ≈1000 (1); >300,000 (1)	Modest overall quality of the studies Studies with longer follow-ups showed a protective association between lactation and the risk of T2DM. The benefit increased with the duration of lactation and was independent of other T2DM risk factors. Only one study included in the MA excluded islet autoantibody-positive women
**Feng L et al., 2018 [17]** SR and MA of cohort studies To investigate the association between lactation and the development of T2DM in women with prior GDM.	Medline Embase and Cochrane Library From inception to June 2017 Search terms provided English language only	Thirteen studies. All included in the MA All from high-income countries All cohort	GDM had various diagnostic criteria Different BF definitions used among studies A total of 8/13 studies were adjusted for confounders	**BF vs. no-BF** BF reduced the risk of T2DM compared to no-BF **Thirteen studies pooled** BF significantly associated with a 34% lower risk of T2DM RR = 0.66, 95% CI 0.48–0.90, *p* = 0.008 **Eight adjusted studies only** RR = 0.69 (0.50–0.94), *p* = 0.018 **Six prospective studies only** RR 0.56 95% CI (0.41–0.76), *p* < 0.001 **Four retrospective studies only** RR 0.63 95% CI (0.40–0.99), *p* = 0.044 **Duration of lactation** **Three studies** BF duration of 4 to 12 weeks, compared to no lactation, was not associated with T2DM risk: OR = 0.69, 95% CI 0.41–1.17, *I*^2^ = 84.4%, *p* < 0.050 The F/U of the included studies were 1.5 to 3 m (5 studies); 1–4 y (4); ≥7 y (4). Sample sizes were 200–500 (3); ≈1000 (1); >300,000 (1)	Moderate- or high-quality studies In subgroup analysis, some studies had characteristics such as follow-up <1 y; only non-adjusted data had non-significant overall effects. Two studies included in the meta-analysis excluded islet autoantibody-positive women
**Ma S et al., 2018 [18]** SR and MA of observational studies To provide comprehensive analyses of current research developments in the field of BF and metabolic-related outcomes among women with prior GDM.	Medline, Embase, BIOSIS Previews, Web of Science and Cochrane Library From inception to Dec 2017 Search terms provided No language restriction	Twenty-seven studies Twenty-five from high-income and two from upper-middle-income countries	GDM various diagnostic criteria BF status and intensity. measured at discharge or 4–14 weeks pp; length varied between studies. Seven studies correctly adjusted covariables	**Duration of lactation** **Overall** **Fifteen studies (9290 women)** Lower risk of T2DM among women with longer BF (definition varies from article to article); any intensity OR 0.79, 95% CI 0.68–0.92, (*p* = 0.002) *I*^2^ = 33.3%, *p* = 0.090 **Stratified by follow-up period:** Eight cohorts * 1–6 m: OR = 0.93 95% CI 0.52–1.67 (*p* = 0.800), *I*^2^ = 54.9%, *p* = 0.030 **Three cohorts** 1–5 y: OR = 0.67, 95% CI 0.47–0.96 (*p* = 0.028) *I*^2^ = 0.80%, *p* = 0.365 **Seven cohorts** >5 y: OR = 0.81, 95% CI 0.72–0.90 (*p* < 0.001) *I*^2^ = 17.2%, *p* = 0.299 **Twelve cohorts** OR 0.77; 95% CI 0.67, 0.89, (*p* < 0.001) **Five cross-sectional studies** OR 1.15; 95% CI 0.52, 2.55, (*p* = 0.723) **Lactation intensity** **Two studies** Full BF vs. non-BF was protective of T2DM, evaluated at 1–5 y pp OR 0.53 95% CI 0.29–0.95; *p* = 0.033	Pooled results of cross-sectional studies only did not show the effect of longer BF Two studies included in the meta-analysis excluded islet autoantibody-positive women
**Tanase-Nakao K et al., 2017 [19]** SR and MA of observational studies To review the current findings on lactation for T2DM prevention in women with previous DGM.	Medline, CINAHL, Embase From inception to Dec 2015 Search terms provided No language or time restriction	Fourteen reports from nine studies All from high-income countries Five cohorts and four cross-sectional	GDM various diagnostic criteria or self-report BF, intensity at 6–9 weeks postpartum and/or duration Data used for the MA were crude and unadjusted for covariables.	**Duration of lactation** **Overall** **Five studies** (3408 women) (Quality of evidence: low to very low) A BF duration longer than 4 to 12 weeks, compared to shorter duration, had a different risk reduction for T2DM OR 0.29, 95% CI 0.14–0.58; (*p* < 0.01) *I*^2^ = 85%, *p* < 0.01 **Stratified by follow-up period:** **Two cohorts** <2 ** y OR = 0.77, 95% CI 0.01–55.86; (*p* = 0.91) *I*^2^ = 89.0%, *p* = 0.003 **One cohort** 2–5 y OR = 0.56, 95% CI 0.35–0.89; (*p* = 0.02) *I*^2^ = NA **Two cohorts** >5 y OR = 0.22, 95% CI 0.13–0.36; (*p* < 0.01) *I*^2^ = 85.0%, *p* = 0.03 **Lactation Intensity** **One study** (1035 women) (Quality of evidence: moderate) Exclusive lactation for 6–9 weeks was associated with 58% lower risk of T2DM, OR 0.42 95% CI 0.22–0.81	Conclusions on T2DM incidence for each study: 6 studies reported results in favor, and 3 reported null results The positive effect of lactation was not obvious when diabetes was evaluated in the early postpartum period; it became clear with longer follow-up. Only the studies evaluating T2DM after 2y pp excluded islet autoantibody-positive or early-onset DM.

BF—breastfeeding; no-BF—no breastfeeding; CV—cardiovascular; CINAHL: cumulative index to nursing and allied health literature; IADPSG—international association of diabetes in pregnancy study groups; SR—systematic review; MA—meta-analysis; m—months; F/U—follow-up; y—years; GDM—gestational diabetes mellitus; T2DM—type 2 diabetes mellitus; RR—relative risk; OR—odds ratio; CI—confidence interval; *I*^2^: heterogeneity index; NA—not applicable; pp—postpartum. * A study may have been analyzed in more than one cohort. ** The authors grouped the short F/U group as <2 y; however, upon revising the original articles, the real FU was <3 m.
